# Prevention of Preeclampsia

**DOI:** 10.1155/2012/435090

**Published:** 2012-12-17

**Authors:** Sammya Bezerra Maia e Holanda Moura, Laudelino Marques Lopes, Padma Murthi, Fabricio da Silva Costa

**Affiliations:** ^1^Department of Public Health, State University of Ceara, 60.740-000 Fortaleza, CE, Brazil; ^2^Division of Maternal-Fetal Medicine, Department of Obstetrics and Gynaecology, Schulich School of Medicine & Dentistry, University of Western Ontario, London, ON, Canada N6H 5W9; ^3^Department of Perinatal Medicine, Pregnancy Research Centre, The Royal Women's Hospital, 7th Floor, 20 Flemington Road, Parkville, Melbourne, VIC 3052, Australia; ^4^Department of Obstetrics and Gynaecology, University of Melbourne, Melbourne, VIC 3052, Australia

## Abstract

Preeclampsia (PE) affects around 2–5% of pregnant women. It is a major cause of maternal and perinatal morbidity and mortality. In an attempt to prevent preeclampsia, many strategies based on antenatal care, change in lifestyle, nutritional supplementation, and drugs have been studied. The aim of this paper is to review recent evidence about primary and secondary prevention of preeclampsia.

## 1. Introduction

Preeclampsia (PE) is a multisystem disorder characterized by de novo hypertension and proteinuria or superimposed to maternal hypertension or nephropathy in pregnant women who are usually beyond 20 weeks of gestational age. It affects around 2–5% of pregnancies. The prevalence may range as high as 10 to 18% in some developing countries [1]. PE can be classified into early-onset and late-onset PE and these subtypes may represent different forms of the disease. Early-onset PE is commonly associated with fetal growth restriction (FGR), abnormal uterine and umbilical artery Doppler waveforms, and adverse maternal and neonatal outcomes. In contrast, late-onset PE is mostly associated with milder maternal disease and a lower rate of fetal involvement, with usually favorable perinatal outcomes [2–4].

Screening for PE attempts to identify high-risk pregnancies to modify antenatal care and institute preventive treatment regimens in order to reduce complications and deaths [2, 5–9]. The SCOPE group developed a predictive model for PE based on clinical risk factors for nulliparous women and concluded that screening for PE using maternal history alone is an unreliable method. MAP (mean arterial pressure, defined as “twice the diastolic plus the systolic blood pressure divided by three”) when studied alone in the second trimester of 90 mmHg or above presents a positive likelihood ratio of 3.5 (95% CI 2–5) and a negative likelihood ratio of 0.46 (95% CI 0.16–0.75) to predict PE onset [10]. Howewer, a study combined maternal factors to uterine artery Doppler PI. The detection rate of early PE at a 10% false-positive rate increased from 47% in screening by maternal factors alone to 81% in screening by maternal factors and the lowest UtA-PI. The respective detection rates for late PE increased from 41% to 45% [11]. The concentration of many biomarkers including pregnancy-associated plasma protein A (PAPP-A) and placental growth factor (PIGF) in the maternal serum are known to be altered during the first trimester of pregnancies destined to develop PE [12, 13]. Furthermore, numerous studies have examined the effectiveness of uterine artery Doppler in the second trimester prediction of PE and fetal growth restriction (FGR). This technique has been shown to have controversial effectiveness in terms of such prediction, because the effectiveness is dependent of which outcome would be predicted (early-onset PE, late-onset PE, or FGR) [14]. Recent studies have reported the effectiveness of first trimester uterine Doppler measurements with promising results, mainly in combination with other parameters [15–17]. 

A combined approach has proven to be the best way to increase sensitivity and specificity in many screening tests. Poon et al. [18] proposed a predictive model combining maternal factors, maternal uterine artery Doppler, MAP, PAPP-A, and PlGF. For a 5% false-positive rate, the sensitivity for early-onset PE was 93%. The likelihood ratio for a positive test was 16.5 and the negative likelihood ratio was 0.06. It is the best detection rate published so far, but validation studies are necessary. 

Prevention of preeclampsia may be primary, secondary, or tertiary [8]. Primary prevention involves avoiding pregnancy in women at high risk for PE, modifying lifestyles or improving nutrients intake in whole population in order to decrease the incidence of the disease. Therefore, probably the majority of cases of PE are unpreventable [6]. Secondary prevention is based on interruption of known pathophysiological mechanisms of the disease before its establishment. Recent efforts have focused on the selection of high risk women and have proposed an effective intervention, as early as it is possible, in order to avoid the disease or its severe complications [19]. Tertiary prevention relies on using treatment to avoid PE complications. Magnesium sulfate, for example, is the drug of choice for reducing the rate of eclampsia, but at least 71 women would need to be treated to prevent one case of eclampsia. Therefore, tertiary prevention can be difficult to achieve without exposing many to possibly unnecessary risks [6]. Given the above, the purpose of this paper is to review this recent evidence on the primary and secondary prevention of preeclampsia. 

## 2. Methods 

Literature providing evidence on prevention of preeclampsia published between 2000 and 2011 was assessed in November 2011. Search terms including “preeclampsia,” “prevention,” “prediction,” and “screening” were accessed from Pubmed (MEDLINE), and Cochrane Database of Systematic Reviews. Searches were then supplemented with recommendations from reviews of the bibliographies of other relevant articles and systematic reviews. 

## 3. Importance of PE Prediction 

Identification of women at risk for PE is of major importance for antenatal care. Women identified as high-risk can be scheduled for more intensive antenatal surveillance and prophylactic interventions. Current strategies for risk assessment are based on the obstetric and medical history and clinical examination. Unfortunately, evidence regarding the actual risk associated with individual factors is unreliable [9, 20, 21]. A screening strategy based on maternal history and other risk factors was proposed in the United Kingdom by National Institute of Clinical Excellence (NICE). The results classified more than 60% of pregnant women as high-risk and predicted less than 30% of those destined to develop PE, with a false-positive rate of 10% [22]. 

PE is considered a syndrome, with several presentations, so the ideal predictive test for PE should utilize a combination of many predictors. Since physiopathology of PE considers abnormal placentation and its vascular supply, preexisting maternal subclinical endothelial dysfunctions increase the chances of these phenomena [23]. Poor placentation exacerbates preexisting maternal subclinical disorders including maternal vascular supply and high blood pressure. Due to these assumptions, studies have focused on maternal vasculature in early pregnancy combining maternal history with measurement of blood pressure, uterine artery Doppler, and serum biomarkers for prediction of preeclampsia [9, 11, 13, 15, 18, 21, 24–26]. 

Early detection of PE would allow for planning appropriate monitoring and clinical management, following the early identification of disease complications. Although trials of prophylactic intervention for PE from midgestation have not proven efficacious, it has been suggested that very early prediction of PE in gestation may allow early prophylactic strategies to be more effective [22]. Reliable antenatal identification of PE is crucial to cost-effective allocation of monitoring resources and the use of possible preventative treatment. 

## 4. Importance of PE Prevention 

Many factors complicate the prevention of PE cases. Most are attributed to unknown etiology, the low predictive value of current screening tests, and the several presentations of the disease. Interventions that determine a small reduction in risk mean that a large number of women need to be treated to prevent a single case [27]. For now, definitive treatment remains delivery and removal of the placenta. No effective prophylaxis for PE is formally advised currently. However, given PE is considered such a global health problem, with relatively high rates of maternal and neonatal morbidity and mortality in many countries, prophylactic interventions with small or moderate benefits may be worthwhile. 

## 5. Interventions for PE Prevention 

A number of trials, reviews, and protocols evaluating interventions for prevention of preeclampsia are available in the scientific literature. Current strategies for primary and secondary prevention focus on antenatal surveillance, modification of lifestyle, nutritional supplementation, and pharmacological therapy [27]. Despite the variety of possible prophylactic interventions described, studies have produced disappointing results [24]. Many studies about prevention of PE are based on primary interventions, when applied to whole population : bed rest, restriction of activity or regular exercise, nutritional measures as reduced salt intake, and antioxidants such as vitamins C and E, garlic, marine oil. Other studies are based on secondary prevention, when applied to high-risk population: drugs such as diuretics, progesterone, nitric oxide, calcium supplementation, and aspirin [20]. 

## 6. Rest 

Assuming that exercise would reduce uteroplacental blood flow and bed rest would increase it, as preeclampsia is associated with reduced placental perfusion, bed rest might help prevent this syndrome [28–30]. On the other hand, rest increases risk of deep vein thrombosis and pulmonary embolism, particularly during pregnancy [31]. 

There are few studies about rest in pregnancy. One trial evaluated 32 nulliparous women at 28–32, weeks gestation with normal blood pressure advised to rest at home in left lateral recumbent position for at least 4 hours daily until delivery. There was a statistically significant reduction in the relative risk of PE with four to six hours rest per day (RR 0.05, 95% CI 0.00 to 0.83), but not of gestational hypertension (RR 0.25, 95% CI 0.03 to 2.00), compared to normal activity [32]. Another trial studied 74 primigravida women at 28-29 weeks of gestation with normal blood pressure and a positive roll-over test, as well as MAP at least 80 mmHg. They were advised to rest at home in left lateral position for 15 minutes twice daily, and nutritional supplementation 3 times/week orally with soy protein 25 g, calcium 300 mg, and linoleic acid 300 mg, until delivery. Rest of 30 minutes per day plus nutritional supplementation was associated with a reduction in the risk of preeclampsia (RR 0.13, 95% CI 0.03 to 0.51) and also of gestational hypertension (RR 0.15, 95% CI 0.04 to 0.63) [33]. Higgins et al. [28] reported that women with normal blood pressure in their first pregnancy who were in paid employment had higher blood pressure and higher risk of developing preeclampsia than those who were not in paid employment. 

A Cochrane review (2010) included the two small trials (106 women) above besides their uncertain quality and ten other studies were excluded. The review concludes that daily rest, with or without nutrient supplementation, may reduce the risk of preeclampsia for women with normal blood pressure, although the evidence is insufficient to support recommending rest or reduced activity to women for preventing PE and its complications [30]. 

## 7. Exercise or Other Physical Activity 

Reduction in the risk of hypertension in nonpregnant patients by regular exercise and physical activity was considered as successful strategy, therefore it was proposed that exercise and physical activity may help prevent preeclampsia. It is important to evaluate whether exercise reduces the risk of PE and its complications and if these possible benefits outweigh the risks. Observational studies of regular recreational physical activity during pregnancy report a reduced risk of preeclampsia [34], therefore it was suggested that exercise may even help to prevent preeclampsia [35]. However, at present there is insufficient evidence from randomized trials evaluating aerobic exercise in healthy pregnant women [36] and in women at increased risk of preeclampsia [37]. 

A Cochrane review about this topic examined two small, good-quality trials (involving 45 women). Both compared moderate intensity regular aerobic exercise with maintenance of normal physical activity during pregnancy. Different outcomes were analyzed: preeclampsia (2 trials, 45 women; RR 0.31, 95% CI 0.01 to 7.09); preterm birth (2 trials, 45 women; RR 1.00, 95% CI 0.07 to 13.37); small-for-gestational age babies (1 trial, 16 women; RR 3.00, 95%CI 0.14 to 64.26); caesarean section (1 trial, 29 women; RR 0.93, 95% CI 0.22 to 3.88). Since the trials were too small, there was insufficient evidence to conclude the possible effects of exercise on prevention of PE and its complications [37]. 

## 8. Reduced Dietary Salt 

The advice of reducing salt during pregnancy is a common practice among clinicians, probably because this is a valid recommendation for hypertensive patients in general. A Cochrane review published in 2010 compared restricted dietary salt with a normal diet in pregnancy. It included 2 trials, with 603 women as participants of the study. However, there was no significant correlation observed (RR 1.11, 95% CI 0.46 to 2.66) to advice reduced salt intake during pregnancy [38]. 

## 9. Garlic 

There are suggestions that garlic may lower blood pressure in a nonpregnant population. A meta-analysis of 8 trials (415 participants) reported reductions in both systolic and diastolic blood pressure are associated with garlic treatment in the form of dried powder [39]. Experimental studies have demonstrated that garlic inhibits platelet aggregation [40] and may also increase the production of nitric oxide. Moreover, it was reported that garlic also works as a platelet inhibitor and a vasodilator [41]. These results support the hypothesis that garlic may have a role in the prevention of preeclampsia. 

Extrapolating these data to pregnancy, a trial was performed with 100 primigravida women at moderate risk of PE at 28 to 32 weeks. Use of garlic was compared to placebo. There was no significant difference between the garlic and control groups in the relative risk of gestational hypertension (RR 0.50, 95% CI 0.25 to 1.00) or PE (RR 0.78, 95% CI 0.31 to 1.93). There was no clear difference in other reported side effects [42]. A Cochrane review described this trial to be of uncertain quality and concluded that there is insufficient evidence to recommend increased garlic intake for preventing PE and its complications [43]. 

## 10. Marine Oil 

Marine oil presents hypotensive properties in normotensive and hypertensive nonpregnant women. It influences fatty acids precursors of prostaglandin that modulate inflammatory and vascular effects. Preeclampsia and gestational hypertension are associated with vasoconstriction and endothelial damage, thus marine oil fatty acids could reduce these responses through direct competition with the thromboxane A2 precursor, the arachidonic acid [44]. 

One trial involving 5644 women evaluated a multivitamin and mineral supplement during pregnancy, which included a small amount of fish oil. There was a 31% decrease in preeclampsia with supplementation compared with no supplementation, but it was not possible to conclude that marine oil was the supplement component responsible for the outcomes [45]. A Cochrane review included 6 trials (2755 women) comparing a supplement or food that contained marine fatty acids with either placebo or no treatment. There were no clear differences in the group of high blood pressure (5 trials, 1831 women, RR 1.09, 95% CI 0.90 to 1.33) or the incidence of PE (4 trials, 1683 women, RR 0.86, 95%CI 0.59 to 1.27) between marine oil treatment and control groups. Treatment and control groups did not clearly differ in the incidence of preeclampsia regardless of the timing of supplementation. There is not enough evidence to support the routine use of marine oil or other prostaglandin precursor supplements during pregnancy to reduce the risk of preeclampsia or its complications [46]. 

## 11. Antioxidants 

Some antioxidants such as vitamins C and E act as free radical cleaners. Minerals such as selenium, zinc, and iron are commonly referred to as antioxidant nutrients, but these chemical elements have no antioxidant action themselves and are instead required for the activity of some antioxidant enzymes that act as intracellular defense [47]. Since pregnant women with preeclampsia have decreased plasma and placental concentrations of antioxidants, comes the possibility that placental underperfusion may lead to oxidative stress and an inflammatory response. This would cause inappropriate maternal vascular endothelial cell activation and endothelial cell damage that would result in hypertension and proteinuria. This has led to the proposal that antioxidants may be of benefit as prophylaxis against preeclampsia, by preventing systemic and uteroplacental endothelial damage [48]. 

A Cochrane review included 10 trials and 6533 women. In the majority of trials, the antioxidant regimen assessed was a combination of vitamins C and E therapies. There was no significant difference between antioxidant and control groups for preeclampsia (9 trials, 5446 women, RR 0.73, 95% CI 0.51 to 1.06) or any other primary outcome: severe preeclampsia (2 trials, 2495 women, RR 1.25, 95% CI 0.89 to 1.76), preterm birth before 37 weeks (5 trials, 5198 women, RR 1.10, 95% CI 0.99 to 1.22), small-for-gestational-age infants (5 trials, 5271 babies, RR 0.83, 95% CI 0.62 to 1.11), or any perinatal death (4 trials, 5144 babies, RR 1.12, 95% CI 0.81 to 1.53). Evidence from this review did not support routine antioxidant supplementation during pregnancy to reduce the risk of PE and other complications in pregnancy [49]. 

## 12. Diuretics 

Diuretics are widely used in hypertension in nonpregnant populations. In the past they were used to prevent or delay the development of preeclampsia, based on the supposition that salt intake and retention cause the disease. More recently, evidence shows that women with preeclampsia are hypovolemic and a study showed that women who use diuretics from early pregnancy do not increase their plasma volume as occurs in normal pregnancy [50]. So, suspicions arose that diuretics might worsen the hypovolemia in women with preeclampsia, with adverse effects on the mother and fetus, particularly in terms of fetal growth [2]. 

A Cochrane review included 5 trials comparing thiazide diuretics with either placebo or no treatment (1836 women). There were no clear differences between the diuretic and control groups for any reported pregnancy outcomes including preeclampsia (4 trials, 1391 women, RR 0.68, 95% CI 0.45 to 1.03), perinatal death (5 trials, 1836 women, RR 0.72, 95% CI 0.40 to 1.27), and preterm birth (2 trials, 465 women, RR 0.67, 95% CI 0.32 to 1.41). At present, there is insufficient evidence to provide reliable conclusions about the effects of diuretics on prevention of preeclampsia and its complications. Currently, diuretics should not be recommended for this purpose in routine clinical practice [51]. 

## 13. Progesterone 

Since the 1950s, the hypothesis that progesterone may reduce the risk of preeclampsia has been proposed [52–54]. Progesterone may influence the vascular adaptations of normal pregnancy by decreasing responsiveness of blood vessels to vasoconstrictors and inducing vasodilatation [55, 56]. There is evidence that HLA-G (a protein expressed by the cytotrophoblast) is reduced in the placenta and serum of women with PE [57] and this may contribute to impaired placentation and subsequent preeclampsia. On the other hand, the risk of PE maybe reduced by progesterone in that it enhances the expression of HLA-G protein in placental cytotrophoblast cells [58], which suggests that progesterone may promote immunological tolerance between the fetus and mother. However, progesterone levels were found to be different in primigravida with and without preeclampsia [59]. 

A Cochrane review in 2011 included 4 trials of variable quality (1445 women). Three trials compared women using progesterone injections, and one compared women using progestogen vaginal gel. There were no clear differences between the two groups on risk of preeclampsia (3 trials, 1277 women, RR 1.25, 95% CI 0.95 to 1.63), perinatal death (4 trials, 2594 babies, RR 1.34, 95% CI 0.78 to 2.31), preterm birth (3 trials, 1313 women, RR 1.01, 95% CI 0.93 to 1.10), small-for-gestational-age (1 trial, 168 babies, RR 0.82, 95% CI 0.19 to 3.57), major congenital defects (3 trials, 2436 babies, RR 1.19, 95% CI 0.31 to 4.52), or any other outcome reported. There were no reported cases of masculinization of female babies (1 trial, 128 women). The authors concluded that there is insufficient evidence for reliable conclusions about the effects of progesterone for preventing preeclampsia and its complications. Therefore, progesterone should not be used for this purpose in routine clinical practice at present [60]. 

## 14. Nitric Oxide 

The pathophysiology of PE involves a maternal vascular endothelial cell dysfunction. Vasodilatation and inhibition of platelet aggregation are some functions of endothelium derived nitric oxide. Drugs that enhance nitric oxide levels are nitric oxide donors such as glyceryl trinitrate, isosorbide mononitrate, isosorbide dinitrate, S-nitroglutathione, and sodium nitroprusside. Such drugs are used to treat diseases such as angina and hypertension. Nitric oxide synthase are a family of enzymes that catalyze the production of nitric oxide from its precursor, an aminoacid, L-arginine. Nitric oxide signaling is mainly mediated by the guanylate cyclase/cyclic guanylate monophosphate pathway. The effects of this second messenger system are limited by enzymatic degradation through phosphodiesterases. Drugs such as tadalafil, sildenafil, and vardenafil that are inhibitors of phosphodiesterases enzyme are widely used to treat male sexual impotence and pulmonary hypertension [61]. 

During normal pregnancy, nitric oxide contributes to physiological vasodilatation, decreased responsiveness to vasopressors, and increased uteroplacental blood flow. In preeclampsia, availability of nitric oxide is reduced, but it is unclear whether there is reduced production or increased degradation [61]. There are contradictory results among therapeutic agents that increase nitric oxide, or nitric oxide donors and precursors, to prevent or treat PE, but some studies have demonstrated that administration of nitric oxide donors is associated with a reduction in uterine artery resistance in women with PE [61]. This suggests that nitric oxide may have a role in prevention and treatment of preeclampsia. 

A Cochrane review (2007) included 6 trials (310 women). Four trials were of good quality and two were of uncertain quality. Four trials (170 women) compared nitric oxide donors (glyceryl trinitrate) or precursors (L-arginine) with either placebo or no intervention. There are insufficient data for reliable conclusions about the effects on preeclampsia (4 trials, 170 women, RR 0.83, 95% CI 0.49 to 1.41) or its complications. One trial (36 women) compared a nitric oxide donor with Nifedipine, and another (76 women) compared it with antiplatelet agents. Both were too small for reliable conclusions about possible differential effects. Glyceryl trinitrate was associated with an increased risk of headache (2 trials, 56 women, RR 6.85, 95%, CI 1.42 to 33.04), and of stopping treatment (2 trials, 56 women, RR 4.02, 95% CI 1.15 to 14.09) compared to placebo. However, the increase for both outcomes was due to an extreme result in one small trial (7/7 versus 0/9 for both outcomes). As the sample sizes of trials were small, the authors concluded that there is insufficient evidence whether nitric oxide donors and precursors prevent preeclampsia or its complications [61]. 

## 15. Calcium 

Pregnant women with high levels of calcium intake, such as Guatemala Indians and Ethiopians have a low incidence of preeclampsia and eclampsia [62, 63]. Such data support the hypothesis that an increase in calcium intake during pregnancy might reduce the incidence of high blood pressure and PE among women with low calcium intake. More recent studies have confirmed the association between PE and hypocalcemia [64] and hypocalciuria [65]. 

It has been proposed that low-calcium intake may increase blood pressure by stimulating either parathyroid hormone or renin release, increasing intracellular calcium in vascular smooth muscle and leading to vasoconstriction [66]. Calcium supplementation may reduce parathyroid release and could reduce smooth muscle contractility. It could also reduce uterine smooth muscle contractility or increase serum magnesium levels and thus prevent preterm labour and delivery [67, 68]. Recently, a lower resistance index by Doppler in uterine and umbilical arteries in pregnant women with calcium supplementation has been demonstrated [69]. 

A Cochrane review including 3 studies of good quality (15730 women) was published in 2010. The average risk of high blood pressure was reduced with calcium supplementation rather than placebo (12 trials, 15470 women, RR 0.65, 95% CI 0.53 to 0.81). There was also a reduction in the average risk of preeclampsia associated with calcium supplementation (13 trials, 15730 women, RR 0.45, 95% CI 0.31 to 0.65). The effect was greatest for women with low baseline calcium intake (8 trials, 10678 women, RR 0.36, 95% CI 0.20 to 0.65) and those selected as being at high risk (5 trials, 587 women, RR 0.22, 95% CI 0.12 to 0.42). The average risk of preterm birth was reduced in the calcium group overall (11 trials, 15275 women, RR 0.76, 95% CI 0.60 to 0.97) and amongst women at high risk of developing preeclampsia recruited to 4 small trials (568 women, RR 0.45, 95% CI 0.24 to 0.83). There was no overall effect on the risk of stillbirth or death before discharge from hospital (11 trials 15665 babies, RR 0.90, 95% CI 0.74 to 1.09). Either maternal death or serious morbidity was reduced (four trials, 9732 women, RR 0.80, 95% CI 0.65 to 0.97). There was no statistically significant difference about maternal death in calcium and placebo group, (RR 0.17, 95% CI 0.02 to 1.39). The authors concluded that calcium supplementation appears to approximately reduce in a half the risk of preeclampsia, preterm birth, death, or serious morbidity, especially in high risk women with previous low-calcium intake. There were no other clear benefits or harms [70]. 

Calcium supplementation in the second half of pregnancy seems to reduce blood pressure directly, but does not prevent the endothelial damage associated with preeclampsia [71]. 

## 16. Antiplatelet Agents 

During placental development trophoblastic invasion of the spiral arteries occurs from 8 to 16–20 weeks of gestation. Defective placentation leads to inadequate uteroplacental blood perfusion and ischemia, resulting in maternal vascular to endothelial dysfunction, with platelet and clotting system activation. These issues support the hypothesis that antiplatelet agents might prevent preeclampsia and FGR [72, 73]. Low-dose aspirin could inhibit thromboxane-mediated vasoconstriction, prevent failure of physiological spiral artery transformation and, thus, minimize development of preeclampsia and FGR [74, 75]. In the first trials, treatment started relatively late in pregnancy (after 18 to 20 weeks) and some of them included low-risk patients, resulting in no evidence of benefit [76, 77]. Researchers wondered if better results could be obtained with earlier treatment directed at high risk groups. 

A meta-analysis including all randomized trials (5 studies) evaluated the effectiveness of aspirin compared with placebo or no treatment in women with an abnormal uterine artery Doppler and clinically relevant perinatal and maternal outcomes. There was a significant benefit of aspirin in reducing preeclampsia (OR 0.55, 95% CI 0.32 to 0.95). The baseline risk of PE in women with abnormal uterine artery Doppler was 16%, and the number of women needed to be treated with aspirin to prevent one case of preeclampsia was 16 (95% CI 8 to 316). Women on aspirin had babies who were on average 82 g heavier than controls, but this result did not reach statistical significance (weighted mean difference 81.5, 95% CI 40.27 to 203.27). It was suggested that aspirin therapy in women with abnormal uterine artery Doppler was accompanied by a significant reduction in rates of PE, but not a decrease in FGR [78]. Abnormal Doppler of uterine arteries is well established as a predictor of preeclampsia and FGR, but it is performed most times after 20 weeks of gestation, when the pathophysiological mechanisms of PE are already established [79]. More recent trials evaluated Doppler ultrasound of uterine arteries in thefirst trimester, trying to predict preeclampsia (especially early-onset preeclampsia). If this is successful, it may also be possible to institute earlier therapies, with better results, before the onset of endothelial damage [80–82].

A Cochrane review (2007) containing 59 trials (37560 women) reported a 17% reduction in the risk of PE associated with the use of antiplatelet agents (46 trials, 32,891 women, RR 0.83, 95% CI 0.77 to 0.89). Although there was no statistical difference in RR based on maternal risk, there was a significant increase in the absolute risk reduction of PE for high risk compared with moderate risk women. Antiplatelet agents were associated with an 8% reduction in the relative risk of preterm birth (29 trials, 31,151 women, RR 0.92, 95% CI 0.88 to 0.97), a 14% reduction in fetal or neonatal deaths (40 trials, 33,098 women, RR 0.86, 95% CI 0.76 to 0.98), and a 10% reduction in small-for-gestational age babies (36 trials, 23,638 women, RR 0.90, 95% CI 0.83 to 0.98). There were no statistically significant differences between treatment and control groups for any other outcomes. It was concluded that antiplatelet agents, largely low-dose aspirin, have moderate benefits when used for prevention of PE and its consequences [83]. 

A meta-analysis evaluated the stratification of treatment at various gestational ages to improve the results of aspirin low-dose treatment in healthy, nulliparous pregnant women. They assessed 9 randomized controlled trials with a total of 1317 women meeting the inclusion criteria. Aspirin beginning in early gestation was associated with a greater reduction in the incidence of PE than treatment beginning in late gestation. Treatment started at 16 weeks gestation resulted in reduction in the cases of PE with (RR 0.48; 95% CI 0.33 to 0.68), at 17–19 weeks (RR 0.55; 95% CI 0.17 to 1.76), and at 20 weeks (RR 0.82; 95% CI 0.62 to 1.09). Aspirin treatment started before 16 weeks was also linked with a significant reduction in the incidence of severe PE (RR 0.10; 95% CI 0.01 to 0.74), gestational hypertension (RR 0.31; 95% CI 0.13 to 0.78), and IUGR (RR 0.51; 95% CI 0.28 to 0.92). It was concluded that aspirin treatment initiated early in pregnancy is an efficient method of reducing the incidence of PE and its consequences in women with ultrasound evidence of abnormal placentation diagnosed by uterine artery Doppler [84].

A more recent meta-analysis aimed to estimate the effect of low-dose aspirin in women at moderate or high risk for preeclampsia. The analysis included 27 studies (11348 women) with followup for the outcome of preeclampsia. Low-dose aspirin started at 16 weeks or earlier was associated with a significant reduction in PE (RR 0.47; 95% CI 0.34 to 0.65, prevalence in 9.3% treated compared with 21.3% control) and FGR (RR 0.44; 95% CI 0.30–0.65, prevalence in 7% treated compared with 16.3% control), whereas aspirin started after 16 weeks does not produce the same results (PE: RR 0.81, 95% CI 0.63–1.03, prevalence in 7.3% treated compared with 8.1% control; FGR: RR 0.98, 95% CI 0.87–1.10, 10.3% treated compared with 10.5% control). Low-dose aspirin started at 16 weeks or earlier also was associated with a reduction in severe PE (RR 0.09, 95% CI 0.02–0.37, 0.7% treated compared with 15.0% control), gestational hypertension (RR 0.62, 95% CI 0.45–0.84, 16.7% treated compared with 29.7% control), and preterm birth (RR 0.22, 95% CI 0.10–0.49, prevalence 3.5% treated compared with 16.9% control). These studies concluded that low-dose aspirin initiated in early pregnancy is an effective method of reducing the incidence of PE and FGR in high and moderate risk women [85]. 

Prevention of preeclampsia is more likely to be successful by identifying women at high risk and scheduling them to proper antenatal care. Efforts should be made to find earlier PE predictors in order to institute interventions before 16 weeks [86, 87]. 

## 17. Conclusions 

Interventions such as rest, exercise, reduced salt intake, garlic, marine oil, antioxidants, progesterone, diuretics, and nitric oxide showed insufficient evidence to be recommended as preventive measurements for PE. On the other hand, low-dose aspirin especially when initiated before 16 weeks in high-risk groups, and calcium especially in low-intake populations show promise in the prevention of PE. The results of large clinical trials in high-risk populations selected during the first trimester of pregnancy are keenly awaited. 
